# Race and Biomedicine Beyond the Lab: 21st Century Mobilisations of Genetics—Introduction to the Special Issue

**DOI:** 10.1057/s41292-021-00261-5

**Published:** 2021-10-27

**Authors:** Anne Pollock, Amade M’charek, Nadine Ehlers, Melissa Creary, Vivette García-Deister

**Affiliations:** 1 Department of Global Health and Social Medicine, King’s College London, London, UK; 2 Department of Anthropology, University of Amsterdam, Amsterdam, The Netherlands; 3 Department Sociology and Social Policy, University of Sydney, Sydney, Australia; 4 School Public Health, University of Michigan, Ann Arbor, USA; 5 Faculty of Sciences, National Autonomous University of Mexico, Mexico, Mexico

Genetic knowledge is paradigmatically laboratory knowledge, and yet its power does not rise or fall on scientific truth claims situated there. This is perhaps less obvious in cases where genetic knowledge circulates within the realm of science. However, nowadays, when genetic knowledge has left the laboratories to play an increasingly important role in everyday life (in the form of direct-to-consumer tests, genealogical research, forensics, etc.), the question of how and where knowledge is co-produced and how this affects the lives of people merits more attention. Take the example of race. If the persistent sociological debunking of race, indicating that it is an illegitimate scientific category, fails to undermine the social power of race, that might spur us to ground our analysis elsewhere.

This Special Issue investigates a range of objects and cases to show the value of moving our analysis of race and biomedicine beyond the lab. Specifically, we ask, how are genetic ideas of race taken up, deployed, and potentially reworked outside the laboratory environment in instances or events such as a Mayan ceremony at a human rights forensic genetics lab in Guatemala; a grassroots Race Against Blood Cancers recruitment drive by and for ethnic minorities in the UK; a fair-skinned mother with sickle cell trait mobilising “legitimate suffering” in a patient-activist group in Brazil; a US lawsuit claiming that a “mixed-race” child born from a sperm donor mix-up was “wrongfully born”; the complicated relationships with Chinese-ness in population genetics projects in Vietnam and Singapore; the pursuit of an unknown Turkish suspect in the Dutch policing system? What do these diverse stories tell us about genetics? And how do they advance our thinking about the complicated relations between genetics and race?

This Special Issue is a project from “Race and Biomedicine Beyond the Lab”, a group initially drawn together around a Wellcome Trust small grant and continuing as a loose international research network called “RaBBL” (rather affectionately pronounced “rabble”). There are two key decentring moves that guide both the network and the papers in this volume. First, as the title of this Special Issue gives away, moving beyond the lab is to decentre the lab as a privileged site of knowledge-making. Without denying the lab’s continuing relevance or power, papers in this volume foreground interferences (Haraway 1991) of different kinds of knowledges that do not contribute to homogeneity, attending to tensions and differences between sites of production. Second, debates on race have been dominated by US practices and cases, thus generating specific modes of knowing race and racism. Through the geographically widespread cases we attend to here, we also want to decentre this US approach by making space to consider other dynamics and mobilisations of race (again without denying the power and relevance of US approaches). We do this through foregrounding ways that race *operates* across a diverse geo-political range and through exploring multidimensional ways race is put to *use*.

Begun in 2019 with a single in-person conference, before the pandemic made such gatherings impossible, the papers have been developed in a context where our initial questions of concern have become matters of much larger interest. In 2020, the intertwined global phenomena of the crisis of COVID-19 and the resurgence of Black Lives Matter have brought increasing attention to racial health disparities. The confluence has provoked increasing recognition of the inseparability of the social and the biological, amid recognition of the role of political injustice in structuring the unequal impact of Covid, broader social disparities, and police violence. Within a short amount of time in the last couple of years, *racism* has taken centre stage and has come to be far more widely recognised as a pervasive (and enduring) problem in science and society. Prominent institutions such as the US Centers for Disease Control have declared that “racism is a serious public health threat” (CDC 2021). This marks overdue institutional acknowledgement of antiracist public health scholarship and activism (Airhihenbuwa and Ford 2018; Jones 2000). But that recognition is not resolution. In this moment, there are many vital ways that biomedical truth claims and practices are being mobilised to both shape and contest contemporary race and racism. The time is ripe for the elaboration of empirically grounded justice-oriented engagement with race as an object of biomedicine that is not contained within the lab or the clinic.

This introduction takes the reader along the journey to the RaBBL intervention, that is, decentring dominant stories of race and biomedicine. After providing a background on the critical scholarship of race and biomedicine, we describe how biomedical ideas are being mobilised beyond the lab. We then turn to the specific topic of this Special Issue, briefly tracing out the interrelations between genetic research and race. Following this, we introduce the papers in this Special Issue, each of which explore how such ideas have moved beyond the lab into the social realm and the political issues at stake.

## Race and biomedicine, in the lab and beyond

How are we to study race and its associated relations of power, what sociologist Howard Winant (2015, p. 313) has evocatively termed the “‘dark matter’ of the modern epoch”, as they intersect with biomedicine? Neither race nor biomedicine is a stable or singular object that precedes our ability to analyse them. Race is an historical product (Ehlers 2012; Fields 1982) that is both unstable (Hammonds and Herzig 2009) and tenacious (Wade et al. 2014). It is not a singular or a metaphysical entity. Processes of racialisation have been shown to be contradictory practices (Erasmus 2017; M’charek 2013). As Amade M’charek argues (2013, p. 424), “[t]he challenge in studying race is to denaturalize without dematerializing it, and to simultaneously attend to materiality without fixing race”. Biomedicine is also diffuse and shifting. It operates differently in alternative temporal moments and spheres and produces divergent forms of knowledge (Bliss 2012; Roberts 2011). In emergent knowledges and practices of biomedicine, racial difference is a “durable preoccupation” (Pollock 2012), and race is “enacted” as it is stabilised and silenced (M’charek 2013; Williams 2018). Inevitably, the relationship between race and biomedicine is fraught.

### Race in science, medicine, and technology

A rich body of literature exists on race in the biosciences, biomedicine, and technology. Much of this work might be seen as exploring and challenging the operations of what philosopher Charles Mills calls “the Racial Contract”: that set of formal and nonformal agreements that have structured the creation of racial hierarchy and the founding of a racial polity (1999, pp. 11–12). As Mills has so powerfully shown, the Racial Contract fundamentally relies on an “epistemology of ignorance” (1999, p. 18) or what he refers to as “a schedule of structural blindnesses and opacities” that have privileged a white polity (1999, p. 19). “[S]ets of perceptions” (Mills 1999, p. 19) are indeed the cornerstone of centuries of scientific positivism—a view that positions objects of study as readily observable and presupposes that scientific knowledge is objective, value-free, and capable of generating universal explanations of “reality”. Investigations of this problematic view structure many critical approaches to the study of race and the biosciences/biomedicine, which seek instead to examine how the creation of racial meaning and “race” as an object of knowledge have never been impartial. We might also see critical literatures on race and biomedicine as directly or indirectly speaking to Michael Omi and Howard Winant’s conceptualisation of “racial projects”: those “historically situated projects in which human bodies and social structures are represented and organized” (1986, p. 56). A racial project, according to Omi and Winant, “is simultaneously an interpretation, representation, or explanation of racial dynamics, and an effort to reorganize and redistribute resources along particular racial lines” (1986, p. 56). In a general sense, scholarship investigating the intersections of race and biomedicine could be seen as tracing how medicine and scientific thought have themselves functioned as particular kinds of racial projects—generating certain racial knowledges and discourses that then become embedded within social practices and structures.

As Katrina Karkazis and Rebecca Jordan-Young (2020, p. 765) have recently noted, this critical work on the intersections of race and biomedicine, the biosciences, and technology has taken two primary trajectories. In the first trajectory, we see efforts to identify how race has been understood in different historical periods—to “pin down what race ‘actually is’” (what it *means*) in specific moments (Rajagopalan et al. 2016, p. 349)—through examinations of *genealogies of the race idea in the biosciences and biomedicine*. For example, numerous studies chart histories of race and racial classification—as they are enmeshed in the biosciences and biomedicine—in relation to colonial expansion, European settlement, slavery, and nationalism (Anderson 2006; Hammonds and Herzig 2009; Roberts 2011; Rosemblatt 2018; Stepan 1991; Stern 2003; Wade et al. 2014). A further body of work charts post-World War II challenges to the race concept—in light of the crescendo of eugenic science—that sought to decouple the concept of biological race from moral and political association (Gil-Riaño 2018; Lipphardt 2012; Lewontin 2009; Livingston 1962; Montagu 1945; Reardon 2005). And, in the more contemporary context, we see examinations of the geneticisation and molecularisation of race in the biosciences and biomedicine (Bliss 2012; Nelson 2016; Koenig et al. 2008; Whitmarsh and Jones 2010). A second trajectory of scholarship has sought to identify *what race is made to do*: that is, how race functions and how it is operationalised in the context of the biosciences, biomedicine, and contemporary biotechnologies (Benjamin 2014; Braun 2014; Duster 2003; Epstein 2007; Hatch 2016; Kahn 2013; Krupar and Ehlers 2017; M’charek 2020; Nelson 2008; Pollock 2012). In this area of work, race is viewed as concept or indeed a political technology that has been deployed or called on in ways that actualise certain outcomes—whether that be to reinscribe notions of ‘biological difference', structurally created health inequalities, and skewed life-chances, or to problematise the very notion of race and its effects.

The papers in this volume work in this second vein, in that they examine how race—as it intersects with biomedicine—is *mobilised*. For something to be *mobile* it must be able to move or to be moved. Race is mobile in that, rather than being static or fixed, it is capable of meaning different things in different temporal periods and geographical sites or, indeed, in having competing meanings in the same historical or geographical location. It is mobile in that it is a “sliding and ambivalent signifier” (Hall 2017, p. 125). To *mobilise* is an active endeavour, where something is organised or brought into use to achieve a particular goal. Here, we are interested in the various ways that race, in the context of biomedical ideas, rationales, or practices (as a concept, an embodied experience, or identity marker), is brought into use or put to work in order to achieve certain outcomes. These outcomes themselves may be contradictory, as the papers in this collection show, in that some consolidate established regimes of power/knowledge and differential valuations of racial life, while others powerfully contest and seek to alter these same regimes. As noted, however, our focus rests on how race is put to use in various social locales *beyond* the lab.

## Laboratory studies and beyond

In Science and Technology Studies (STS), the *lab* has played a vital role in opening up the black boxes of science. When STS scholars decided to study these sanctuaries of Western knowledge production ethnographically, their key aim was to *locate* the seemingly universal and free-floating knowledge somewhere in place and time. Inspired by the work of Thomas Kuhn and others, these studies showed that science is a *cultural* and thus *specific* practice (Pickering 1992). It is a practice that entails human relations and hierarchies, as well as an elaborate material culture, from instruments and inscription devices to chemical reagents and biological samples; from shoptalk to representational techniques (Knorr-Cetina 1981; Law 1994; Latour and Woolgar 1979; Lynch 1985). Importantly, such studies have also demonstrated that nature is not discovered but *constructed*. First, nature is rendered studiable. Nature is *homed in*, in the words of Karin Knorr-Cetina (1995), so as to become an object of study. How scientists come to know nature thus requires interventions and modifications of, and tinkering with, the object of inquiry. It requires that the object becomes part of the network of knowledge production. Second, extending these networks of knowledge production beyond the lab allows the esoteric knowledge of laboratories to travel to the rest of society and to become *matters of fact*. As Bruno Latour (1988) has shown dramatically in relation to the work of Louis Pasteur, this travel from lab to society requires that society itself changes and complies with the rules of the lab. For example, to make the contraceptive pill effective (that is, scientifically efficacious) is to make people live a regularised life in which women can build in the pill in their everyday rhythm so as not to forget taking it (Oudshoorn 1994).

Labs are situated practices where a specific mode of knowledge production is fostered. And labs are powerful places, not by nature but by building networks so as to make their facts travel. Such networks extend not just within the scientific communities but to various domains in society (institutions, clinics, as well end-users). This, however, does not mean the world is but a mirror image of the lab, or solely a vehicle for laboratory knowledge (e.g. Amsterdamska 1990), precisely because *laboratories are not the only places of knowledge production*. However, the unequal power relation between laboratory science and other forms of knowledge production in general, as well as the power assigned to the lab as a representation of Western knowledge, has contributed to the postcolonial centre–periphery relation between “the West and the rest” (Hall 1992); foregrounding Western knowledge production and trivialising those forms of knowledge developed or staged outside of Western systems and geographies. The study of postcolonial laboratories is one way to challenge this bifurcation (Pollock 2019), and should be complemented with a decolonising move beyond the lab. Indeed, as many have argued, even in critical studies, staging the lab as the key object of critique contributes to the privileging of this site when it comes to knowledge production (Oudshoorn and Pinch 2003). So, moving beyond the lab is a move to decentre the laboratory as the place of knowledge and instead pursue a commitment to decolonise knowledge practices, even if the knowledge at stake is biomedicine or genetics. Moving beyond the lab is not moving outside of laboratory science practices or outside of Western science. Rather, it is an effort to make space for other modes of knowing and for the interferences between different knowledge practices.

While laboratory studies have undoubtedly contributed to our understanding of how race is produced through scientific practice (Bliss 2013; García-Deister 2014; Fullwiley 2008; M’charek 2005; Muniz 2019), in this Special Issue, we take up the challenge of considering how race and biomedicine are constantly *reconfigured* outside the walls of laboratories. As noted above, we do not see this reconfiguration as unidirectional (from lab out to society). Instead, we focus on the ways bioscientific ideas operate in relation to plural genealogies of race in particular contexts. In order to focus on mobilisations of race and biomedicine beyond the lab, we take a distinctive approach that is rooted in postcolonial science studies, critical race studies, and intersectional feminism: we engage with race as an unstable and contested object of inquiry, seek to make scientific and medical research and our own scholarship accountable to justice, and understand all knowledges as necessarily partial and situated (Haraway 1988). Our approach here is global and multi‐sited. Our aim, however, is not to produce one universal answer to matters of race, racialisation, or racism. In contrast, we collectively aim to counter the homogenisation of meanings, claims, and practices across a range of sites of inquiry.

## Genetics

From the wide-ranging topics at the RaBBL conference in 2019, we selected just one topical thread to workshop for development into this Special Issue, namely *genetics*. While this volume is not intended as a definitive compilation, but instead a starting point, there are good reasons to begin with genetics and racialisation—and how bioscientific understandings of the relation between race and genetics travel outside the lab. Genetics indeed offers a vital site for intervention into the scholarly terrain because in the scholarship of race and science broadly, genetics is often a key focal point for engagement—whether debunking genetic claims or exploring the generativity of genetic thinking—and so engaging genetics engages the core of the field and seeks to move it forward.

### Genetics and race

Genetics has been a key field of laboratory science since the late eighties. With the advent of the Human Genome Project, genetics has become almost the synonym for the life sciences (Kay 2000; Keller 2000; Kevles and Hood 1992). While the human genome, the map of the collections of human genes, was presented as a monument of humanity and was said to speak to the communality in our genes, the routes taken in genetic research have foregrounded differences (Duster 2003; Fujimura and Rajagopalan 2010; Hamilton 2008). This focus on difference has without a doubt revitalised biological race (Bliss 2012; Kahn 2013; Lee et al. 2001; M’charek 2020). Over the past decades, we have witnessed the twin politics within genetics of producing knowledge that undermines the existence of biological races (Serre and Pääbo 2004) and mobilising racial categories to make sense of the genetic data; a process that Jonathan Kahn (2012) after Mike Fortun (2008) have called “race in the meantime”. This is to say that geneticists seem to be convinced of the nonexistence of race yet need social categories of difference to order their data and to interpret the results. Some geneticists go further and have recently claimed to have evidence that the results of genetics seem to map onto social clustering and therewith provide evidence for the existence of biological race (Kahn et al. 2018).

The focus on difference in genetic research has famously been conceptualised as the “reinscription of race at the molecular level” (Abu El-Haj 2007) or the “molecularization of race” (Duster 2006; Fullwiley 2008) and it has spurred a large body of scholarship on the social and ethical aspects of genetics/genomics. But this research has also created a sense of urgency to pay closer attention to the fraught relationship between race and biomedicine, a relation that has a complicated historical legacy wherein biomedicine has been part and parcel of a range of social projects. For example, essentialist bio‐scientific ideas of race have circulated widely and have been used historically to justify colonialism, slavery, apartheid, mass genocide, immigration restrictions, and enduring inequality along racial lines (Anderson 2003; Braun et al. 2007; Ehlers 2012; Erasmus 2017). Eugenic beliefs were articulated in official state-sanctioned practice in many countries well into the twenty-first century and continue in what many would call neo‐eugenic forms (Hoffman 2017; Tausig et al. 2003). New epigenetic research does not evade this, but can reinscribe old categories of race, and even as it documents the impact of a racist environment, problematically locate responsibility in the bodies of racialised women (Saldaña-Tejeda 2018; Valdez 2018) in ways that are continuous with medicine’s role in racist medicalisation through both imposition of treatment and denial of care (Edu 2018; Roberts 1997). Race is often poorly defined in biomedical research, yet implicitly relies upon this kind of reification (Lee 2009). The risk with genetic research and its dalliance with race, then, is that it can also revive old ideas of supposed inherent difference in the social realm.

Despite its long‐lasting relation with race and racism, however, biomedicine is ambivalent and can be mobilised for resistance as well: to show the biological effects of racism and structural inequality and to rebut biologically essentialist arguments related to race—in order to support civil rights movements, inclusive citizenship, and various other forms of activism (Creary 2018; Pollock 2012). Because both race and biomedicine are arenas of vital political struggle, neither is the sole prerogative of biomedical and scientific experts (Braun et al. 2007). The tenacity of race requires close attention to the ways its historical use in justifying supremacy has contemporary implications—including but not limited to Black Lives Matter and Covid-19. There is thus a pressing need to examine how race is mobilised—in complicated relations—beyond the lab, in the twenty-first century, and across geographical contexts. Taking up this task in relation to genetics specifically, the papers in this volume ask: how do bioscientific ideas about race and genetics travel into and within the social arena, how are they taken up and deployed in various ways across a range of cultural milieus, and to what ends? The stakes are high in our current global political climate. Shifting beyond the lab to consider mobilisations of race and genetics, what novel knowledges are produced? What modes of articulating difference and injustices are foregrounded?

## Solidarity, resistance, and relatedness: the papers

To understand contemporary mobilisations of race and genetics, the papers in this issue analyse a range of sites beyond the lab, and geographies beyond the US, and reveal race to be at once fixed and unstable, docile and unruly. Noncoherence emerges, then, as a common finding among these writings.

That the embrace of race and/as genetics can be used strategically to mobilise solidarities is most prominent in the first two papers in the issue. This is perhaps most compellingly illustrated in the first paper of the Special Issue, in which Lindsay Smith and co-author Vivette García-Deister draw on ethnographic research in multiple Latin American sites—Argentina, Guatemala, and Mexico—to articulate the concept of “genetic syncretism” in human rights forensics projects. While recognising that forensic genetics reinforces racial ideologies that are inextricable from colonial and postcolonial violence, they are also attuned to the simultaneous elements of resistance that can emerge in and through these technologies as they open up pathways for local and global Indigenous (self)recognition and demands for justice.

Recognition and solidarity are also central to Ros Williams’ exploration of Black and Minority Ethnic haematopoietic stem cell donor recruitment drives in the United Kingdom. Williams illuminates how “race and relatedness are mutually enacted in efforts to engage minoritised communities” in these bone barrow donation projects, which aim to construct and mobilise an “ethico-racial imperative” to compel members of these communities to donate. She shows that in order to recruit donors according to notions of genetic relatedness, these nonscientists who are themselves members of racialised minorities engage in deeply affective work, striving to realise relationships of reciprocity by configuring communities as kin.

Yet, sometimes understandings of genetic ties and social solidarities can push in opposite directions. Melissa Creary’s ethnographic engagement with sickle cell disease advocacy organisation in Rio de Janeiro, Brazil, illuminates the problematics of “disrupted biosocial cohesion”. After situating sickle cell disease advocacy in the landscape of transnational biosociality scholarship and grassroots Black activism in Brazil, Creary provides a close reading of powerful but fraught claims to authentic belonging by one activist: a fair-skinned woman with sickle cell trait who is a mother of children who have died of sickle cell disease. Often treated as a leader of the group externally, the activist’s belonging is complicated within, because sickle cell disease is configured not only as a medical diagnosis with serious biomedical consequences but also as a marker of the legitimacy of the social suffering of Afro-Brazilians.

The racialisation of authentic relatedness and belonging is also at stake in the next paper, which analyses another case in which a mother features prominently. Nadine Ehlers’ analysis of the Illinois District Court case *Cramblett vs. Midwest Sperm Bank*, in which a white lesbian mother who gave birth to a black child through a sperm donor mix-up claimed damages of “wrongful birth”. Attuned to the operations of structural racism, biomedicine, and the law in a context of racial capitalism, Ehlers persuasively argues that the loss that the mother claimed can be understood as a loss of “investments in life—and its continuation—as a form of racial property”.

Strategic invocations and denials of relatedness can also take place at larger scales. Tiên-Dung Hà and co-author Mohammad Bin-Khidzer engage in a comparative analysis of two genome projects in Southeast Asia: the Vietnamese Genome Project, run by a private conglomerate, and the Perenakan Genome Project, in Singapore. Charting the projects’ distinctive paths that strategically differentiate from and connect with Chinese identity and nationalism in the context of Chinese regional and global power, Hà and Bin-Khidzer’s comparative approach illuminates how the projects “strategically differentiate and negotiate the ‘bio geo-body’” of Vietnam and Singapore in relationship with the colonial and postcolonial geopolitics.

The downplaying of genetic relatedness and/as race can also be used strategically to resist undermining other solidarities. Irene van Oorschot and Amade M’charek take on a very different legal case, in which police in the Netherlands drew on but downplayed the racialisation of familial DNA in forensic genetics in the pursuit of an unknown suspect. Emphasising the noncoherence of race as it was managed by criminal justice actors involved in the case, van Oorschot and M’charek argue that while forensic genetics can reproduce racial understandings of human difference, those mobilising the technology in this case employed complicated manoeuvers of care, taming, and erasure to “keep race at bay”.

Finally, the Special Issue also features a Books Forum, co-authored by Vivette García-Deister and Anne Pollock, that explores bleak biopolitics and abolitionist aspirations in a set of six recent books on race: *Silent Cells: The Secret Drugging of Captive America* by Anthony Ryan Hatch (2019); *Deadly Biocultures: The Ethics of Life-Making* by Nadine Ehlers and Shiloh Krupar (2019); *Beneath the Surface: A Transnational History of Skin Lighteners* by Lynn Thomas (2020); *Race Otherwise: Forging a New Humanism for South Africa* by Zimitri Erasmus (2017); *Becoming Human: Matter and Meaning in an Antiblack* World by Zakiyyah Iman Jackson (2020); and *Captivating Technology: Race, Carceral Technoscience*, and the *Liberatory Imagination* edited by Ruha Benjamin (2019).

Taken together, the issue offers a corpus of beyond-the-lab studies that illustrates multiple directions that biosocial analyses of race have taken. Extending and complicating existing epistemologies of race and genetics, RaBBL offers a set of provocations for the field.
